# Wisdom Tooth Autotransplantation for the Missing Maxillary Central Incisors Using a 3D-Printed Replica: A Case Report

**DOI:** 10.7759/cureus.61327

**Published:** 2024-05-29

**Authors:** Yuji Haneda, Takeshi Murayama, Hiroki Nikawa, Saiji Shimoe, Masato Kaku

**Affiliations:** 1 Dentistry, Yutori Dental Clinic, Tokyo, JPN; 2 Medical System Engineering, Division of Oral Health Sciences, Hiroshima University, Graduate School of Biomedical Sciences, Hiroshima, JPN; 3 Oral Biology and Engineering, Division of Oral Health Sciences, Hiroshima University, Graduate School of Biomedical Sciences, Hiroshima, JPN; 4 Anatomy and Functional Restorations, Division of Oral Health Sciences, Hiroshima University, Graduate School of Biomedical Sciences, Hiroshima, JPN

**Keywords:** central incisor, wisdom tooth, teeth missing, trauma, autotransplantation

## Abstract

In this case report, we describe a 19-year-old man who underwent an autotransplantation of a lower third molar into the extracted region of his upper central incisors. Due to trauma, the patient’s upper right and left central incisors had been extracted. He visited our clinic and requested to perform autotransplantation of his own teeth into the upper central incisor part because he wanted to use his natural teeth. So, we decided to extract his lower right third molar and autotransplant it into the extraction part of the upper central incisors.

Immediately after extraction of the lower right third molar, the tooth was autotransplanted into the upper anterior region using a 3D-printed resin replica of the donor tooth and artificial sockets of the recipient site. Then, the root canal treatment was performed, and a temporary crown was set. Next, orthodontic treatment was done to flatten the curve of Spee. After completing the orthodontic treatment, a final prosthodontic restoration was set on the autotransplanted tooth. Four years later, the autotransplanted tooth remained stable with a healthy periodontium. This case demonstrates that if a patient has a request to use their natural teeth, autotransplantation of a wisdom tooth into the anterior region can be a useful method to replace the missing teeth.

## Introduction

Tooth autotransplantation is a valuable and frequently applied technique for replacing tooth loss due to its good and reliable outcomes. The key success factor of tooth autotransplantation is healthy periodontal ligament (PDL) tissues on the donor's teeth because the PDL has osteoinductive ability, which can promote alveolar bone regeneration [1,2]. For autotransplantation into the anterior region, both upper and lower premolars are suitable, showing more than a 96% success rate 10 years after the operation [3]. On the other hand, an unnecessary third molar is used as a donor tooth for replacing a missing molar, and the survival rates for mature third molar autotransplantation are reported to be more than 90% [4]. However, there is no report of third molar autotransplantation into the anterior region because it is challenging due to the complex root anatomy of a donor tooth and bone volume of the recipient site. This case report shows a third molar autotransplantation into the upper anterior region using a 3D-printed resin replica of the donor's tooth and artificial sockets of the recipient site. As a result, acceptable aesthetic and functional occlusion was achieved without defective dental restorations.

## Case presentation

Pre-treatment evaluation

**Figure 1:**
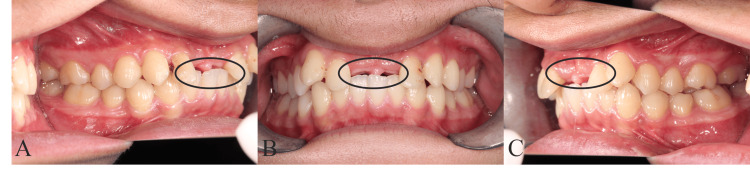
Pre-treatment intraoral photographs (A) The right side. (B) The front view. (C) The left side. The encircled region indicates the missing upper right and left central incisors.

**Figure 2:**
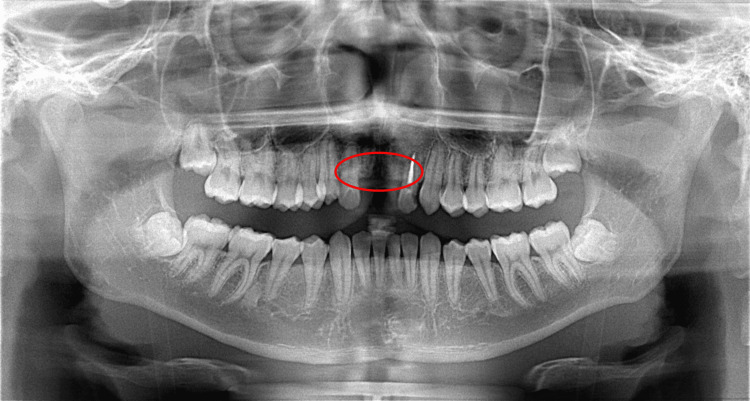
Pre-treatment panoramic radiograph The bone volume of the missing incisor area was reduced (indicated by the encircled region).

The patient is a 19-year-old male with a chief complaint of missing teeth on both the upper right and left central incisors. He got injured in a traffic accident three years before and set a removable partial denture. The pre-treatment facial photograph, initial intraoral photographs, and panoramic radiograph revealed loss of the upper central incisors (Figures 1, 2).

The possible treatment approach was as follows. The first option was replacement of the space by a dental implant, and the second was setting a fixed partial denture from the upper right canine to the left canine. We recommended these two treatment options; however, the patient rejected our opinion and asked us to use the third molar for autotransplantation. We explained to him that third molar autotransplantation is challenging due to the root and crown size of the donor tooth and the bone volume of the recipient site for both esthetic and stability. Informed consent for autotransplantation was given to the patient and his parents before the start of treatment, and they selected this option.

Treatment plan for the lower right first molar

The treatment plan was as follows. First, the patient was received professional tooth brushing instruction before autotransplantation. After taking cone-beam computed tomography (CBCT), a 3D-printed resin replica of the donor's tooth was made, and pre-planning autotransplantation with artificial sockets of the recipient site was performed. After that, the lower right third molar was extracted and autotransplanted into the upper anterior region. Then, the curve of Spee was flattened, and the upper anterior crowding was treated by orthodontic tooth movement. Finally, the autotransplanted tooth was restored by prosthodontic restoration.

Treatment progress

**Figure 3:**
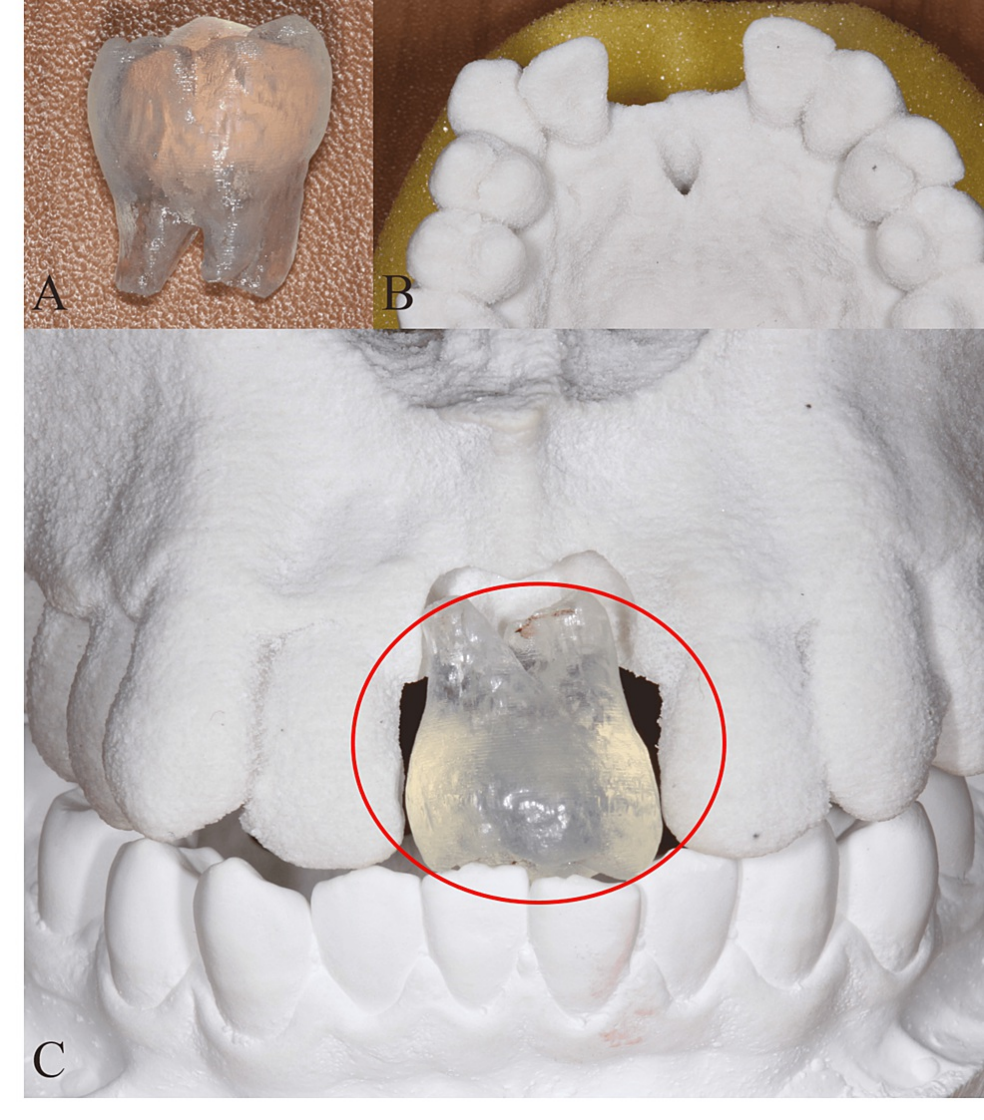
The 3D-printed teeth and maxilla replica model (A) The 3D-printed replica of the lower right third molar. (B) The plaster model of the maxilla. (C) The encircled region shows the adjustment of the 3D-printed replica tooth into the recipient site of the plaster model.

After taking the CBCT radiograph, a 3D-printed replica of the lower right third molar and the plaster model of the maxilla and mandible were made by JMC (JMC Corporation, Yokohama, Japan). The 3D-printed replica tooth was adjusted to the recipient site of the plaster model before autotransplantation (Figure 3).

**Figure 4:**
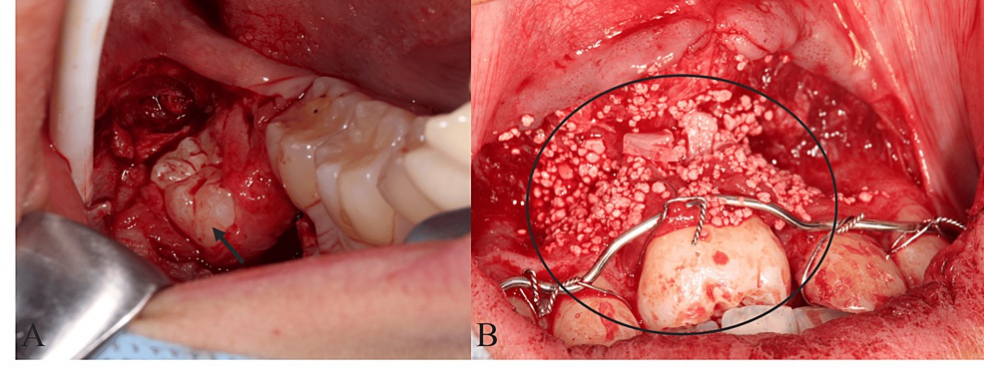
The third molar extraction for autotransplantation (A) The arrow shows the third molar before autotransplantation. (B) The encircled region shows the area immediately after transplantation of the donor tooth and autogenous bone and β-TCP. TCP: Tricalcium phosphate.

Under local anesthesia, the lower right third molar was carefully extracted by extraction forceps (Figure 4, Panel A). After extraction of the lower right third molar, the extracted donor tooth was put into the original alveolar socket. Then, the socket preparation of the recipient site was performed according to the 3D model. The extracted third molar with autogenous bone and β-tricalcium phosphate (β-TCP, Olympus-Terumo Biomaterials, Tokyo, Japan) was transplanted into the sockets immediately and fixed by a wire (Figure 4, Panel B).

**Figure 5:**
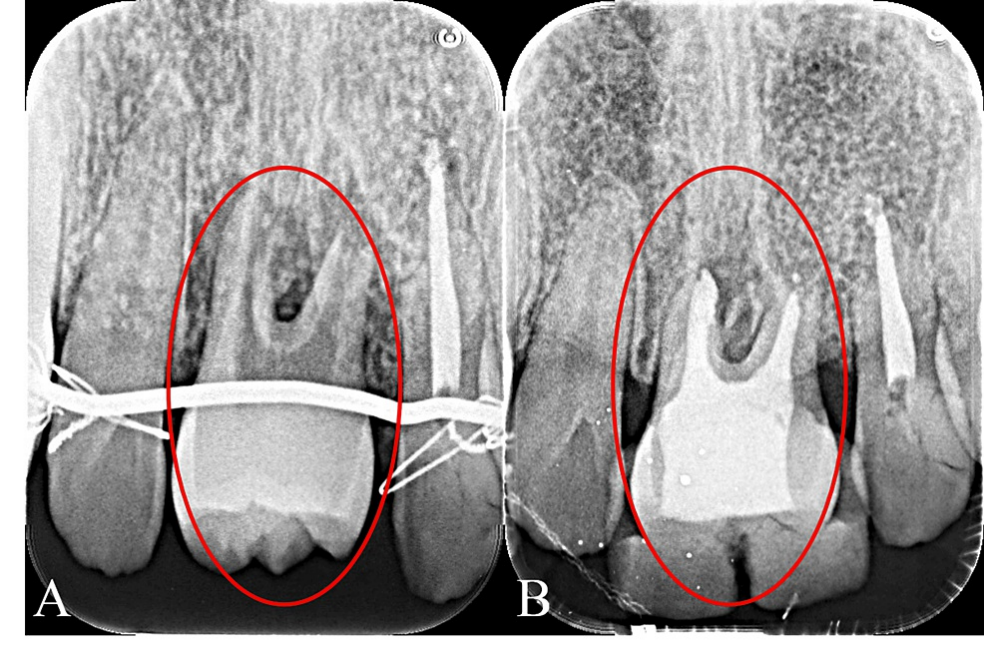
Dental X-ray after autotransplantation (A) Immediately after autotransplantation. (B) After root canal treatment. The circle shows the autotransplanted tooth.

The dental X-ray immediately after autotransplantation showed a good fitting to the alveolar socket (Figure 5, Panel A). Then, endodontic treatment and the root canal filling with gutta-percha point and sealer were performed (Figure 5, Panel B).

**Figure 6:**
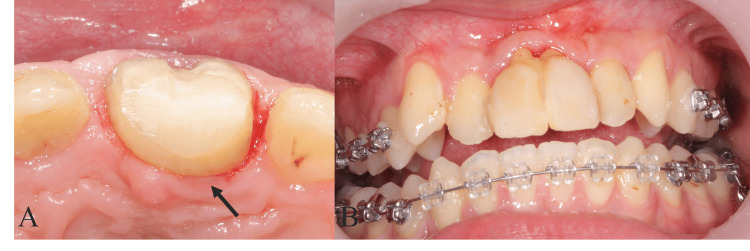
Intraoral photograph of setting the temporary crown for upper central incisors (A) The abutment preparation for the autotransplanted tooth. The arrow shows the autotransplanted tooth. (B) The intraoral photograph of the beginning of orthodontic treatment.

Then, abutment preparation for the autotransplanted tooth was performed, and the temporary crown was set on the autotransplanted tooth (Figure 6, Panel A). After that, flattening the curve of Spee and leveling of upper dental arch by orthodontic treatment were performed to eliminate excessive occlusal load to the autotransplanted tooth (Figure 6, Panel B).

**Figure 7:**
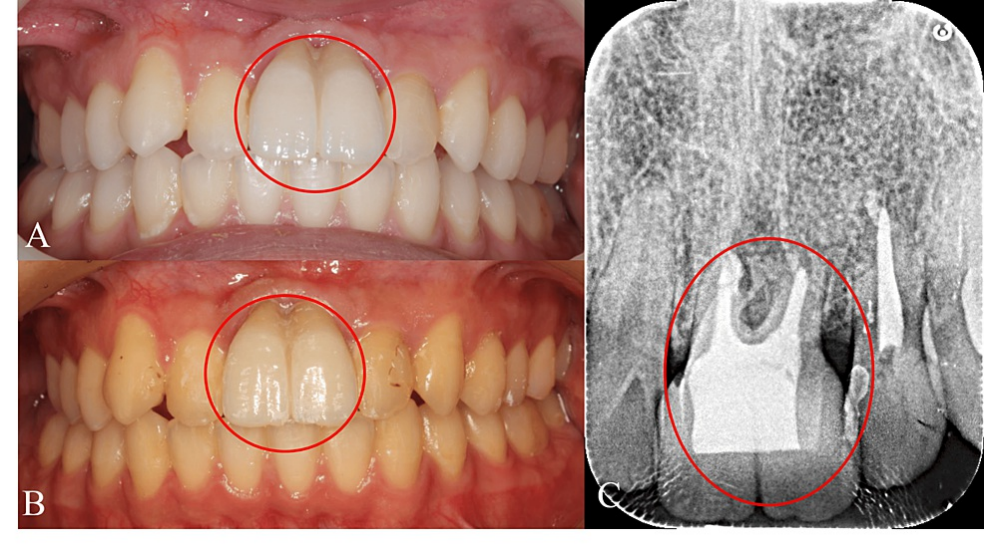
Intraoral photograph after setting a final prosthodontic restoration (A) Immediately after setting a final prosthodontic restoration. (B) The intraoral photograph taken four years after autotransplantation. (C) Dental X-ray taken four years after autotransplantation. The encircled region shows the autotransplanted tooth.

After orthodontic treatment, a final prosthodontic restoration was set on the autotransplanted tooth (Figure 7, Panel A). The autotransplanted tooth is healthy and stable even four years after autotransplantation (Figure 7, Panel B). The dental X-ray taken four years after autotransplantation revealed preservation of alveolar bone height, and inflammatory root resorption of the autotransplanted tooth was not observed (Figure 7, Panel C).

## Discussion

In order to cover replacing missing teeth, several treatments such as fixed dental bridges, removable partial dentures, or dental implants are performed. The greatest advantage of dental implants is that abutment preparation is not necessary for adjacent teeth. However, this patient was a young 19-year-old man. As it was reported that the morphology of the maxillofacial region will change even after adulthood [5], it is possible that the incisor height and the gingival margin in this patient may change in the future and aesthetic problems might occur [6]. Moreover, the patient entreated us to apply the third molar autotransplantation to replace the missing upper anterior teeth. That is why we did not select a dental implant for the defective restorations in this case.

Autotransplantation is a conventional and useful treatment for replacing missing teeth because of a long-term good prognosis. It was reported that the survival rate after autotransplantation was approximately 90% [7]. Especially, in the anterior region, both upper and lower premolars that were extracted due to an orthodontic treatment are often used. Louropoulou et al. showed that the survival and success rate of immature premolar autotransplantation was 99.8% after 10 years of observation. In adolescents, the 10-year survival and success rate of mature premolars were 100% and 96.3%, respectively. The 10-year survival and success rate in adults were 87.5% [3]. Thus, premolar autotransplantation to the anterior region is thought to be a very reliable procedure with a long-term prognosis. The third molar is used as a donor tooth for replacing a missing molar. It is also reported that there were no significant differences in the survival rates for mature third molar autotransplantation into molar fresh extraction sockets and surgically created sockets with guided bone regeneration (93.1% and 95.2%, respectively) [4].

In the present case, the patient lost his upper anterior right and left central incisors due to a traffic accident. As the extraction sockets had already filled with alveolar bone during the three years, it was thought to be difficult to transplant the third molar into the upper anterior region. So, we applied pre-planning autotransplantation using a 3D-printed resin replica of the donor tooth and artificial sockets of the recipient site. It was reported that the total operation time in a 3D replica group was significantly reduced compared to a control group, showing that 3D printing technology can increase the treatment effects of autotransplantation [8]. Also, in this case, 3D-assisted surgery helped to fit the complicated donor roots into the recipient site. Then, as the patient had the deep curve of Spee, flattening the lower dental arch was performed by orthodontic treatment to distribute masticatory forces. Moreover, the patient received professional tooth brushing instructions for dental plaque removal to maintain good oral hygiene including the autotransplanted tooth [9,10].

In the present case, inflammatory root resorption and replacement resorption were not observed, and the autotransplanted tooth survived with healthy periodontium four years after surgery. This case report showed that autotransplantation of wisdom teeth into the anterior region can be a useful method to replace the missing teeth if requested by the patient.

## Conclusions

This case report demonstrates an autotransplantation of the lower right third molar into the upper anterior region. Although it was challenging due to the complex root anatomy of a donor tooth and the bone volume of the recipient site, the autotransplantation succeeded by the use of a 3D-printed resin replica of the donor tooth and artificial sockets of the recipient site. The autotransplanted tooth exhibited stable and healthy periodontal tissue four years after the autotransplantation. Thus, if a patient requests, a third molar autotransplantation into the anterior region can be a useful method to replace the missing teeth.
